# The Impact of COVID-19 on Functional Capacity and Pulmonary Outcomes in the Hail Region: A Cross-Sectional Study

**DOI:** 10.3390/jcm13185571

**Published:** 2024-09-20

**Authors:** Omar W. Althomali, Daria Hussain Shaik, Raheela Kanwal, Junaid Amin, Tolgahan Acar, Ahmed Abdelmoniem Ibrahim, Hisham M. Hussein, Aisha Ansari, Ayman A. Alhammad, Mohammad Shahid Ali, Ahmed Alqunun, Wael Alghamdi

**Affiliations:** 1 Department of Physical Therapy, College of Applied Sciences, University of Hai’l, Hail P.O. Box 2240, Saudi Arabia; o.althomali@uoh.edu.sa (O.W.A.); sd.hussain@uoh.edu.sa (D.H.S.); r.sheikh@uoh.edu.sa (R.K.); sj.amin@uoh.edu.sa (J.A.); t.acar@uoh.edu.sa (T.A.); a.abdalmoniem@uoh.edu.sa (A.A.I.); hm.hussein@uoh.edu.sa (H.M.H.); a.ansari@uoh.edu.sa (A.A.); mos.ali@uoh.edu.sa (M.S.A.); 2 Department of Physical Therapy, Cairo University Hospitals, Giza P.O. Box 12613, Egypt; 3 Department of Basic Sciences for Physical Therapy, Faculty of Physical Therapy, Cairo University, Giza P.O. Box 12613, Egypt; 4 Department of Physiotherapy, College of Medical Rehabilitation Sciences, Taibah University, Almadinah Almunawarah P.O. Box 344, Saudi Arabia; aalhamad@taibahu.edu.sa; 5 Akaz Physiotherapy Center, Hail P.O. Box 55436, Saudi Arabia; ahmed.alqunun@hotmail.com; 6 Faculty of Nursing, Nursing Community Health Department, Al-Baha University, Al-Baha P.O. Box 1988, Saudi Arabia

**Keywords:** COVID-19, pulmonary function test, 6 min walk test, 30 s sit-to-stand test, timed up and go test

## Abstract

**Background/Objectives:** COVID-19, caused by the novel coronavirus SARS-CoV-2, has had a significant impact on life worldwide since its emergence in late 2019. The virus has caused a global pandemic, leading to widespread health, social, economic, and psychological effects. COVID-19 mainly affects the respiratory system. This study aimed to compare the functional capacity and pulmonary function outcomes between COVID-19 patients and individuals who have not been infected in the Hail region. **Methods:** Individuals of both genders above 18 years old who had been infected with COVID-19 in the previous 6 months or had never been infected were eligible to participate. Local hospitals and social media apps were used to recruit willing participants. Heart rate, pulse oxygen saturation, blood pressure, pulmonary function test, hand grip strength, and functional tests (6 min walk test, 30 s sit-to-stand test, and timed up and go test) were measured and compared between the groups. Statistical analysis was performed using SPSS Version 25. **Results:** Forty individuals affected with COVID-19 and forty-one healthy individuals were recruited. Our results showed that in individuals affected with COVID-19, scores on the minute ventilation, 30 s sit-to-stand, and 6 min walk tests were significantly lower than among healthy individuals. Other outcomes did not show any statistical differences between the groups. **Conclusion:** This study contributes to a greater understanding of the functional capacity status of individuals with COVID-19. Patients affected by COVID-19 may develop an impaired lung capacity and a decreased function capacity. These factors may negatively affect physical and cognitive health status. Future studies should evaluate the benefits of interventions with rehabilitation exercises following COVID-19. In light of the functional capacity and pulmonary function decline in individuals affected by COVID-19, interventions encompassing pulmonary and functional rehabilitation exercises are recommended to improve physical fitness and pulmonary function post-COVID-19.

## 1. Introduction

Coronavirus disease (COVID-19) was first identified in December 2019, after which the disease spread throughout all parts of China. By February 2020, COVID-19 had spread to numerous other countries [1]. Longitudinal studies have reported that some pathological changes due to COVID-19, including pulmonary fibrosis, atelectasis, muscular weakness, and neuromuscular and psychological disorders, may be due to long periods of bed rest. Furthermore, COVID-19 patients have an impaired quality of life (QoL), which leads to a decreased physical and pulmonary capacity and provides strong evidence for the incorporation of rehabilitation into COVID-19 management [2,3].

Previous studies on the COVID-19 pandemic have revealed that lung function could also be affected in patients with COVID-19 [4]. A study published in The Lancet Respiratory Medicine assessed lung function in COVID-19 survivors in the USA. The research focused on patients who had been hospitalized due to severe symptoms. The study revealed that a significant percentage of patients experienced persistent impairment in lung function, including reduced diffusion capacity and forced vital capacity (FVC), even months after recovery [5]. Patients with COVID-19 are exposed to long-term corticosteroid therapy, which leads to common problems, such as musculoskeletal pain, decreased range of motion, muscular weakness, neuropathy, myopathy, pulmonary dysfunction, dyspnea, confusion, and impaired activities of daily living, all of which can be managed by rehabilitation [6,7]. Strengthening, aerobic, treadmill, and coordination exercises have been recommended for COVID-19 patients [8].

The World Health Organization (WHO) and the Centers for Disease Control and Prevention (CDC) stated that pulmonary rehabilitation, which involves strength training, breathing exercises, and education, can be beneficial for individuals recovering from COVID-19. It aims to improve lung function, reduce symptoms, and enhance overall functional capacity. A prospective observational study in Germany investigated the impact of a multidisciplinary pulmonary rehabilitation approach on COVID-19 patients. The results indicated improvements in lung function and reduced anxiety levels among participants [9]. Rehabilitation programs are typically tailored to individual needs and may involve a multidisciplinary approach with input from respiratory therapists, physiotherapists, and other healthcare professionals [10].

Although most people make a full recovery from COVID-19, some can develop complications. COVID-19 rehabilitation focuses on helping people regain their physical, psychological, and mental abilities after the recovery period [11]. COVID-19 mainly affects the respiratory system, with effects primarily attributed to heightened immune response activity and inflammatory damage to organs; however, mid- and long-term evidence does not indicate continuing organ dysfunction [12]. Evidence from previous respiratory syndromes, namely severe acute respiratory syndrome (SARS) and Middle East respiratory syndrome (MERS), shows that pulmonary function and capacity to exercise, although initially reduced, improve with time, as do radiological abnormalities [13]. However, this improvement may be very gradual and may take months or in some cases years [14,15,16].

Interestingly, a recent study investigated the evolution of musculoskeletal symptoms in long COVID-19 syndrome [17]. Long COVID-19 syndrome can be defined as a condition in which previously affected individuals experience new, persistent, or returning symptoms. The study highlighted the progressive growth of weakness and fatigue over time and the association of arthralgia with long COVID-19 syndrome. The authors concluded that an interdisciplinary management approach is needed and that long COVID-19 syndrome can be identified as a dynamic entity that requires up-to-date rehabilitation. Ongoing testing of patients with COVID-19 has provided data on pulmonary function after the impact of the virus. It is important to note that the understanding of COVID-19 and its long-term impacts is still evolving, and researchers are actively studying its effects on functional capacity and pulmonary outcomes. Therefore, the aim of this study was to compare pulmonary function outcomes and functional capacity outcomes among COVID-19 patients and non-affected individuals in the Hail region.

## 2. Materials and Methods

The current study was conducted at University of Hai’l in Hail, Saudi Arabia. Ethical approval was obtained from the ethical committees of Hai’l University (H-2021-236) and Hail Health Cluster (2022-6).

### 2.1. Inclusion and Exclusion Criteria

Participants above 18 years of age of both genders were eligible to participate in the study. The control participants were enrolled by posters on the university campus. Participants in the control group had to be healthy and had to have never been affected by COVID-19. The confirmation of no previous infection was performed through TAWAKHALNA application, which is a government application used to track COVID-19 records. The control group participants were excluded if they had any musculoskeletal, neuromuscular, or psychiatric issue affecting walking ability, exercise engagement, muscle function, and active participation in the study.

To be included in the study group, individuals had to have been affected by COVID-19 and subsequently recovered, as confirmed by a positive COVID-19 test. Participants infected with COVID-19 were enrolled by previous hospital records and social media applications with confirmation of the infection within the previous 6 months (1 month to 6 months) via the TAWAKHALNA application. Participants were excluded if they had medical conditions (neuromuscular, musculoskeletal, or psychiatric) that may have affected their muscle function, ability to walk or engage in physical exercise, or active participation in the study. Individuals presently enrolled in or intending to enroll in an alternative rehabilitation program and individuals with any deterioration in physical or psychological well-being were excluded from this study. Lastly, individuals with contraindications related to cardiovascular and respiratory health that precluded engagement in exercise were excluded from the study.

### 2.2. Sample Size

This current study employed convenience sampling to recruit participants.

### 2.3. Outcome Measurement

COVID-19 can lead to respiratory distress and hypoxemia, making it crucial to monitor respiratory function and cardiovascular health and to identify potential complications associated with the disease [18]. In this study, the following outcomes were measured: heart rate, pulse oxygen saturation (SpO2), blood pressure, and pulmonary function test. Pulmonary function was measured using spirometry. The spirometer measures several outcomes, such as forced vital capacity (FVC), forced explanatory volume in the first second (FEV1), and others. FVC can be used to assess the function of the lung. FVC and FEV1 are considered normal when above 80% of the predicted value is achieved [19]. Achieving greater than 70% is considered a mild reduction, 60% to 69% is a moderate reduction, 50% to 59% is a moderately severe reduction, 35% to 49% is a severe reduction, and below 35% is a very severe reduction [20]. The current study extracted FVC, slow vital capacity (SVC), maximal voluntary ventilation (MVV), and minute ventilation (MV). The participants were also subjected to a hand grip strength test (left and right hands) and evaluated based on the distance walked in the 6 min walk test (6MWT), the number of repetitions performed in a 30s sit-to-stand (STS) test, and the timed up and go test (TUG).

Spirometry measures lung function by assessing airflow and volume, serving as a standard diagnostic tool for evaluating lung function and detecting abnormalities like restrictive or obstructive lung diseases. COVID-19 can lead to respiratory symptoms, including shortness of breath and decreased lung function [21]. A Spirovit SP-1 spirometer (Schiller AG, Altgasse, Switzerland) was used for the pulmonary function test, providing accurate and reliable measurements in clinical settings.

Hand grip strength is a measure of upper limb muscle strength and overall muscle function. Although not directly related to pulmonary function, hand grip strength can provide insights into overall physical health and functional capacity. COVID-19 can lead to muscle weakness and fatigue, making grip strength a relevant outcome measure for assessing physical recovery and functional status [22]. A hydraulic hand dynamometer, which is a reliable and valid instrument, was used to measure grip strength [23].

The 6 min walk test provides reliable information about cardiovascular and pulmonary function, as well as overall physical endurance [24]. COVID-19 can cause fatigue, dyspnea (shortness of breath), and reduced exercise tolerance. The sit-to-stand test measures lower limb strength and functional mobility by counting the repetitions of STS movements in 30 s, making it relevant for assessing muscle strength in COVID-19 patients [25]. Additionally, the timed up and go test is useful for evaluating functional mobility and balance, as COVID-19 can impact these areas [26].

### 2.4. Procedure

**Figure 1:**
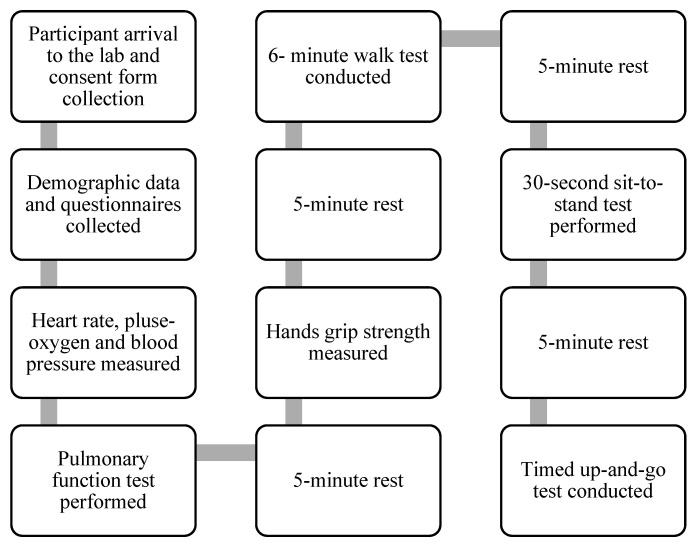
Summary of the data collection procedure.

The data collection procedure is summarized in Figure 1. Upon arrival, demographic information was collected, and heart rate, pulse, oxygen saturation, and blood pressure were measured while the participants were seated. Before the main tests, participants were given practice time for each assessment. During the pulmonary function test, participants used a spirometer while seated, employing a sterile mouthpiece for inhalation and exhalation following the manufacturing manual. They were monitored for any breathing difficulties, dizziness, or other issues.

Hand grip strength was measured using a hand-held dynamometer (Jamar Hydraulic hand dynamometer), with three trials for each hand. The 6 min walk test involved participants walking as quickly as possible in a straight line for 6 minutes, with the option to slow down or stop if fatigued. The distance covered was measured at the end of the test. For the sit-to-stand test, participants used a standardized 43 cm-high chair without arms, standing fully with knee extension and sitting back down with their hands crossed over their chest within 30 s. If they could not complete a trial, their score was zero, and an adapted test was used. The timed up and go test required participants to sit on a 43 cm-high chair with armrests. They were instructed to stand on command, walk 3 meters, turn around, return to the chair, and sit down as quickly as possible without running. Both groups followed the same procedures for comparison [27].

### 2.5. Statistical Analysis

All data were entered into SPSS Version 25, and statistical analysis was performed. Initially, a normality test was conducted, and if the data were normally distributed, a parametric test was used (independent samples *t*-test); if not, a non-parametric test was used (Mann–Whitney). The significance level was considered 0.05.

## 3. Results

The study recruited 81 participants divided into two groups. The study group consisted of 40 individuals (20 males and 20 females), and the control group comprised 41 individuals (20 males and 21 females). The gender distribution and age range between the study group and the control group are presented in Table 1.

The analysis of the demographic characteristics (age, height, weight, and BMI) and vital signs (heart rate, systolic blood pressure, diastolic blood pressure, and respiratory rate) revealed no significant differences between the groups, as shown in Table 2.

There were no significant differences between the groups regarding the SpO2, FVC, SVC, MVV, left-hand strength, right-hand strength, and TUG. The analysis revealed significant differences in MV, STS, and the 6MWT between the groups. The control group exhibited a higher MV compared to the study group. The mean MV in the study group was 16.38 L/min (SD = 10.07), whereas in the control group, it was 41.87 L/min (SD = 36.25). This difference was found to be statistically significant (*p* < 0.05) based on statistical tests (independent *t*-tests). Additionally, the study group demonstrated significantly better performance on the STS test compared to the control group. The mean number of successful STS repetitions in the study group was 11.48 (SD = 2.81), whereas in the control group, it was 10.22 (SD = 2.55). This difference was found to be statistically significant (*p* < 0.05). Furthermore, the control group showed a better performance in the 6MWT compared to the study group. The mean distance covered during the 6MWT in the study group was 653.32 m (SD = 183.32), while in the control group, it was 747.23 m (SD = 221.11). This difference was found to be statistically significant (*p* < 0.05) (Table 3).

## 4. Discussion

Limited information is available concerning the enduring consequences of a SARS-CoV-2 infection in individuals who have recovered from COVID-19 [28]. The current study aimed to investigate the effect of COVID-19 on pulmonary function and functional outcomes. Our findings indicate a noteworthy reduction in minute ventilation and performance on the 30 s sit-to-stand test and 6 min walk test among those who had experienced COVID-19 compared to a control group.

Numerous studies examining patients during follow up after recovering from COVID-19 have consistently identified diminished functional capacity [29,30,31]. It is interesting to note that evidence from prior coronavirus outbreaks documents impaired pulmonary and physical function, emotional distress, and diminished QoL among survivors [4]. This decline in function and the perception of a deteriorated QoL may be attributed to persistent symptoms, muscle weakness, reduced pulmonary function, and physical deconditioning in these individuals [32]. Interestingly, even though they had severe complications post-COVID-19, a significant proportion of the participants had successfully resumed their work responsibilities within three to four months of hospital discharge [33,34,35].

A recent study aimed to examine the functional status of patients during the subacute phase of hospitalization for COVID-19. All patients received medical treatments along with mobilization and/or physiotherapy at the bedside. The researchers documented improvements in all functional domains of interest following physiotherapy; however, a significant proportion of these patients were discharged with impaired functional capabilities [36]. The mechanisms contributing to low physical functioning and impaired daily activities in COVID-19 survivors include muscle weakness due to deconditioning, respiratory complications, neurological impacts, psychological factors, such as anxiety and depression, and persistent systemic inflammation. These findings lend support to the notion that the lingering effects of COVID-19, referred to as COVID-19 sequelae, may endure well beyond the hospitalization period, thereby potentially impacting various aspects of functional independence among affected individuals.

Numerous potential mechanisms have been implicated in the sequelae of COVID-19. Among these, the enduring respiratory repercussions of SARS-CoV-2 infection remain a subject of ongoing debate. Drawing from previous studies on the SARS pandemic, it has been postulated that lung function can also be affected in patients with COVID-19 [4,37,38]. Furthermore, a recent review by Cagnazzo et al. highlighted that as many as 21.3% of COVID-19 patients exhibited neurological symptoms. Skeletal muscle injury (observed in 5.1% of patients) and dizziness (noted in 1.3%), two of the most commonly recorded neurological manifestations, may also influence physical performance in the long term [39].

Our study revealed that patients with COVID-19 covered a significantly shorter distance in the 6MWT than individuals in the control group. Consistent with our findings, this effect may become more pronounced in patients with post-COVID-19 conditions that exhibit clinically significant differences [40]. Moreover, this impact might be heightened in individuals who have experienced prolonged bed rest and are older, as these could potentially influence balance and gait patterns and strategies [4]. Indeed, recent research has indicated that as many as 70% of post-COVID-19 patients continued to exhibit values below the estimated 6 min walk distance one year after their hospital discharge [39].

Furthermore, our study demonstrated that individuals who had been infected with COVID-19 performed significantly less on the STS test than the control group. Notably, a previous study reported that while 83% of post-COVID-19 patients were able to complete this test, 90% scored below the 25th percentile relative to their baseline values [41]. Furthermore, 32% of patients experienced a decline in pulse oxygen saturation of four points or more during the 1 min sit-to-stand test. Consequently, the 1 min sit-to-stand test proved effective in distinguishing between individuals who had to undergo a prolonged hospital stay (i.e., stays exceeding 10 days) and those who did not in terms of exertional desaturation [41]. There is a need for further research exploring the underlying mechanisms and the impact of pre-existing functional capacity on the recovery process following COVID-19.

Several studies have highlighted the impact of COVID-19 on patients and their return to work [42,43]. The main challenges are that recovered patients may suffer from long COVID-19 syndrome and the difficulty in diagnosing such a syndrome [43]. A previous study showed that such individuals may need special occupational rehabilitation to facilitate their return to work. Supporting evidence showed that the return-to-work time rate was later among patients with COVID-19 compared to flu-like symptoms, and that their return-to-work time rate shortened over time [42]. However, the study assessed only the return-to-work time rate and did not assess the presence of symptoms. Several factors were found to affect the return-to-work time rate, such as age, gender, risk factors, symptoms of fatigue, and shortness of breath. Another challenge that may face individuals suffering from long COVID-19 syndrome is the awareness of society and employees. These individuals suffer from fatigue, reduction in cognitive function, sleep disturbance, and deterioration in work performance [42], which may affect them psychologically and reduce their QoL.

In future research endeavors, it is imperative to conduct focused evaluations to elucidate the potential influence of these specific factors on the physical function of COVID-19 survivors. Additionally, it is crucial to acknowledge that the COVID-19 pandemic compelled people to adhere to stay-at-home measures, resulting in a significant reduction in their physical activity and the adoption of highly sedentary lifestyles. This behavioral shift can have a profound impact on musculoskeletal health, even among individuals not directly afflicted by the virus [44]. Considering rehabilitation, it is essential to underscore that owing to the urgency of the pandemic, an appropriate rehabilitation paradigm was not consistently applied following COVID-19 infection.

Interestingly, the age distribution of the participants in the current study is relatively young. The mean age of 31.59 years in the study group and 36.33 years in the control group. This raises concerns about the representativeness of the sample, particularly for older adults who are more likely to experience severe COVID-19 symptoms and prolonged recovery times [45,46]. Consequently, the findings may not be generalizable to older populations, who may exhibit different recovery trajectories and functional impairments. To address this limitation, future studies should aim to include a more diverse age range by increasing the sample size and ensuring adequate representation of older adults. Targeted recruitment strategies in settings that primarily serve older populations, such as nursing homes or geriatric clinics, could enhance the robustness of the findings and provide a more comprehensive understanding of the long-term effects of COVID-19 across various age groups.

Our study is subject to several limitations that should be acknowledged. A significant limitation pertains to our patient selection process, as we exclusively reached out to individuals who had been infected with COVID-19 severe enough to visit the hospital for a COVID-19 check. This could introduce a selection bias. Furthermore, the current study used a convenient sampling technique, which has the advantage of being easy and quick, it but may reduce the generalizability of the results. In a broader context, more extensive investigations with larger sample sizes are imperative to assess in depth the potential enduring effects of COVID-19 on individuals’ capacity to return to work and other outcomes. Additionally, one limitation of our study is the potential influence of gender and age group (elderly population) on recovery outcomes; however, we believe that these factors do not significantly affect the overall results, as the improvements documented were consistent across diverse patient profiles, indicating that the observed benefits of rehabilitation apply broadly regardless of these demographic variations. Although this sample size may be adequate for exploratory analysis, it limits the capacity to identify subtle differences and perform detailed subgroup analyses. Future research should aim to increase the sample size and diversify the participant demographics to strengthen the findings, gain a deeper understanding of the long-term effects of COVID-19 across various groups, and detect subtle differences.

## 5. Conclusions

The current study demonstrates that a significant proportion of COVID-19 survivors continue to experience persistent symptoms, including diminished functional capacity and pulmonary issues. These findings highlight the long-term impact of COVID-19 and the critical role of ongoing care. To optimize recovery and improve long-term outcomes, it is recommended to implement pulmonary and functional rehabilitation programs. Additionally, the use of post-COVID profiles, such as those based on the minute ventilation, 30 s sit-to-stand test, and 6 min walk test, can be used as valuable tools for assessing functional status, identifying patients at risk for complications, and monitoring recovery progress. By incorporating these strategies, healthcare providers can offer comprehensive care to post-COVID patients and enhance their quality of life.

## Figures and Tables

**Table 1:** Demographic characteristics of study participants, including age range and gender distribution in the study group and control group.

Variable	Study		Control	
	Male	Female	Male	Female
Age (mean (SD))—Years	40.00 (11.40)	22.75 (5.52)	41.26 (8.47)	31.86 (13.00)
(Maximum–Minimum)—Years	(64–26)	(45–18)	(57–24)	(74–20)
(18–36 years) Counts	10	19	8	14
(37–55 years) Counts	9	1	11	6
(56–74 years) Counts	1	0	1	1

**Table 2:** Demographic characteristics and vital signs in the study and control groups.

Variable (Unit)	Study Group Mean (SD)	Control Group Mean (SD)	*p*-Value	Mean Difference	SD Error Difference	95% Confidence Interval of the Difference		Effect Size Cohen’s d
						Lower	Upper	
Age (Year)	31.59 (12.48)	36.33 (11.94)	0.08	−4.74	2.71	−10.14	0.66	0.39
Height (cm)	167.15 (10.12)	167.68 (8.93)	0.80	−0.53	2.12	−4.75	3.70	0.06
Weight (kg)	72.57 (18.01)	78.02 (16.62)	0.16	−5.45	3.85	−13.12	2.22	0.31
BMI (kg/m^2^)	25.71 (4.66)	27.61 (4.92)	0.08	−1.90	1.06	−4.02	0.21	0.40
Heart rate (beats per minute)	78.29 (9.78)	78.38 (11.58)	0.97	−0.08	2.38	−4.82	4.65	0.01
Blood pressure, systolic (mmHg)	118.41 (14.12)	119.30 (19.25)	0.81	−0.89	3.74	−8.34	6.57	0.05
Blood pressure, diastolic (mmHg)	76.78 (9.10)	79.55 (13.27)	0.28	−2.77	2.52	−7.79	2.25	0.24
Respiratory rate (breaths per minute)	17.76 (3.01)	18.50 (2.35)	0.22	−0.74	0.60	−1.94	0.45	0.28

**Table 3:** Main study outcomes in the study group and control group.

Variable (Unit)	Study Group Mean (SD)	Control Group Mean (SD)	*p*-Value	Mean Difference	SD Error Difference	95% Confidence Interval of the Difference		Effect Size Cohen’s d
						Lower	Upper	
SPO_2_: pulse oxygen saturation (%)	96.83 (1.28)	96.93 (1.53)	0.76	−0.10	0.31	−0.72	0.53	0.07
FVC: forced vital capacity (liters)	2.59 (0.93)	2.87 (0.88)	0.17	−0.28	0.20	−0.68	0.12	0.31
SVC: slow vital capacity (liters)	3.5 (2.3)	3.4 (1.6)	0.78	0.12	0.45	−0.77	1.01	0.06
MVV: Maximal voluntary ventilation (L/min)	77.87 (45.39)	76.79 (42.25)	0.91	1.07	9.75	−18.33	20.48	0.02
MV: Minute ventilation (L/min)	16.38 (10.07)	41.87 (36.25)	**>0.01**	−25.49	5.88	−37.19	−13.78	0.96
Right hand strength (kg)	27.57 (7.17)	26.14 (7.96)	0.40	1.44	1.68	−1.91	4.79	0.19
Left hand strength (kg)	27.20 (8.15)	25.13 (7.52)	0.24	2.08	1.74	−1.39	5.55	0.26
STSP: 30 s sit-to-stand (repetition per 30 s)	10.22 (2.55)	11.48 (2.81)	**0.04**	−1.26	0.60	−2.44	−0.07	0.47
TUG: timed up and go test (seconds)	13.38 (6.25)	14.20 (7.01)	0.58	−0.83	1.48	−3.76	2.11	0.12
6MWT: 6 min walk test (meters)	653.32 (183.32)	746.23 (221.11)	**0.04**	−92.91	45.08	−182.64	−3.17	0.46

Bold is significant.

## Data Availability

The raw data supporting the conclusions of this article will be made available by the authors on request.
